# Circular RNA hsa_circ_0000282 contributes to osteosarcoma cell proliferation by regulating miR-192/XIAP axis

**DOI:** 10.1186/s12885-020-07515-8

**Published:** 2020-10-23

**Authors:** Houkun Li, Limin He, Yuan Tuo, Yansheng Huang, Bing Qian

**Affiliations:** Department of Spine Surgery, Honghui Hospital, Xi’an Jiaotong University, No. 76 Nanguo Road, Xi’an, 710054 Shaanxi China

**Keywords:** Osteosarcoma, circ_0000282, miR-192, XIAP, Proliferation, Apoptosis

## Abstract

**Background:**

Circular RNAs (circRNAs) have emerged as a novel category of non-coding RNA, which exhibit a pivotal effect on regulating gene expression and biological functions, yet how circRNAs function in osteosarcoma (OSA) still demands further investigation. This study aimed at probing into the function of hsa_circ_0000282 in OSA.

**Methods:**

The expressions of circ_0000282 and miR-192 in OSA tissues and cell lines were examined by quantitative real-time polymerase chain reaction (qRT-PCR), and the correlation between the expression level of circ_0000282 and clinicopathological features of OSA patients was analyzed. The expressions of X-linked inhibitor of apoptosis protein (XIAP), B-cell lymphoma-2 (Bcl-2) and Bcl-2 associated X protein (Bax) in OSA cells were assayed by Western blot. The proliferation and apoptosis of OSA cells were examined by CCK-8, BrdU and flow cytometry, respectively. Bioinformatics analysis, dual-luciferase reporter gene assay and RIP experiments were employed to predict and validate the targeting relationships between circ_0000282 and miR-192, and between miR-192 and XIAP, respectively.

**Results:**

Circ_0000282 was highly expressed in OSA tissues and cell lines, which represented positive correlation with Enneking stage of OSA patients and negative correlation with tumor differentiation degree. In vitro experiments confirmed that overexpression of circ_0000282 markedly facilitated OSA cell proliferation and repressed cancer cell apoptosis in comparison to control group. Besides, knockdown of circ_0000282 repressed OSA cell proliferation and promoted apoptosis. Additionally, the binding relationships between circ_0000282 and miR-192, and between miR-192 and XIAP were validated. Circ_0000282 indirectly up-regulated XIAP expression by adsorbing miR-192, thereby playing a role in promoting cancer in OSA.

**Conclusion:**

Circ_0000282 was a novel oncogenic circRNA in OSA. Circ_0000282/miR-192/XIAP axis regulated OSA cell proliferation apoptosis with competitive endogenous RNA mechanism.

## Background

Osteosarcoma (OSA) is a primary bone malignancy most commonly seen among adolescents, the therapeutic method of which mainly places emphasis on surgical resection in combination with radiotherapy and chemotherapy [1, 2], yet such treatment strategies result in deeply distressing trauma and high disability rate, and the 5-year survival rate is still lower than 60% among patients with metastasis and recurrence [3], implicating the substantial significance of unmasking the underlying mechanism concerned with the tumorigenesis and development of OSA for improving the clinical diagnosis, treatment and prognosis of OSA.

Identified as a novel category of endogenous non-coding RNA, circular RNA (circRNA) presents as a circular structure formed by reverse splicing, thereby possessing greater stability and higher conservation than linear RNA, which endows circRNAs with the potential to be biomarkers for cancers [4, 5]. For instance, circ-ABCB10 is noticeably up-regulated in breast cancer tissues and facilitates breast cancer cell proliferation [6]. Likewise, circ-LDLRAD3 is up-regulated in pancreatic cancer tissues and cell lines, which is closely related to venous invasion, lymphatic invasion and metastasis [7]. It is reported that circ_0032462, circ_0028173, and circ_0005909 are markedly up-regulated in OSA and accelerate the progression of OSA [8]. Circ_0000282 is a member affiliated to circRNA family, and the expression, function and molecular mechanism of circ_0000282 in OSA have not been reported yet.

MicroRNA (miRNA) is characterized as a non-coding RNA consisting of about 20 nucleotides in length, which regulates gene expression at post-transcriptional level [9–11] Additionally, miRNA regulates the proliferation, differentiation, apoptosis and other biological processes of cells by completely or incompletely binding to the 3′-untranslated region (3′-UTR) of mRNA. For instance, miR-491-5p is down-regulated in gastric cancer tissues, and it represses epithelial-mesenchymal transformation of gastric cancer cells by combining 3′-UTRs of SNAIL and FGFR4 [12]. In prostate cancer, augmented miR-153 expression has remarkable association with detrimental prognosis of the patients [13]. Accumulating researches have elucidated that the expression of miR-192 has a reduction in OSA tissues and cells, and it inhibits OSA cell proliferation, metastasis and induces cancer cell apoptosis [14–16], yet the upstream mechanism contributing to decreased expression of miR-192 in OSA remains to be clarified. Interestingly, circRNA can function as a competing endogenous RNA (ceRNA) to inhibit the expression of miRNAs [17–19].

X-linked inhibitor of apoptosis (XIAP) belongs to inhibitor of apoptosis (IAP) family, the members in which share a conservative sequence named baculovirus IAP repeat sequence, which is indispensable for its anti-apoptosis function [20]. Previous studies have demonstrated that XIAP expression is up-regulated in lung cancer, prostate cancer, OSA and other tumors, while its high expression level has relation with unfavorable prognosis of patients [21–23], yet the upstream molecular mechanism leading to XIAP abnormal expression in OSA remains to be elucidated.

Considering circ_0000282 contained potential binding sites with miR-192, we made a scientific hypothesis that circ_0000282 may act as ceRNA to regulate the expression of miR-192 and the progression of OSA. This study confirmed for the first time that circ_0000282 was highly expressed in OSA. It was also demonstrated that circ_0000282 facilitated OSA cell proliferation and impeded cancer cell apoptosis by regulating miR-192/XIAP axis. Our work depicts a novel ceRNA network consisting of circ_0000282, miR-192 and XIAP to explain the progression of OSA, which provides novel clues for the diagnosis and treatment of OSA.

## Methods

### Patients and tissue samples

From OSA cancer patients underwent tumor resection in Honghui Hospital, Xi’an Jiaotong University, 46 pairs of tumor tissues / adjacent normal tissue specimens were available, then stored in liquid nitrogen. All tissues were histologically evaluated and diagnosed as OSA. None of these patients were subjected to chemotherapy, radiotherapy or targeted therapy prior to the surgery. This study was endorsed by the Ethics Committee of Honghui Hospital and strictly followed the Declaration of Helsinki. All patients enrolled signed informed consent.

### Cell culture

Human OSA cell lines (MG-63, U2-OS, 143B and SOSP-9607 cells) and normal osteoblasts (hFOB1.19 cells) were purchased from Chinese Academy of Sciences (Shanghai, China). All cells, except for hFOB1.19 cell line, were cultured in Dulbecco Modified Eagle Medium (DMEM) (Invitrogen, Carlsbad, CA, USA), and 10% fetal bovine serum (FBS) (Gibco, Grand Island, NY, USA), 100 U/mL penicillin, 100 μg/mL streptomycin (Sigma, St. Louis, MO, USA) were supplemented. hFOB1.19 cells were cultured in Dulbecco’s Modified Eagle’s Medium/Nutrient Mixture F-12 (DMEM/F-12), supplemented with 10% FBS (Gibco, Grand Island, NY, USA). The cell were cultured at 37 °C in 5% CO_2_. Cells in the logarithmic growth phase were selected for subsequent experiments.

### Cell transfection

MiR-192 mimics, miR-192 inhibitors (miR-192 in), pcDNA3.1-circ_0000282, small interference RNAs (siRNAs) targeting circ_ 0000282 (si-circ_0000282) and their negative controls were available from GenePharma (Shanghai, China). The cells were inoculated on a 24-well cell culture plate with 3 × 10^5^ cells/well at 37 °C in 5% CO_2_ for 24 h, and then cell transfection was carried out. When the cell confluency reached 70 to 80%, in accordance with the manufacturer’s instructions, oligonucleotides and plasmids were transfected into 143B cells and MG-63 cells at a final concentration of 50 nM using Lipofectamine®^3000^ (Invitrogen, Carlsbad, CA, USA). 48 h later, the cells were collected for subsequent tests.

### Quantitative real-time polymerase chain reaction (qRT-PCR)

Total RNA in tissues and cell lines was extracted with RNA isolation kit (Invitrogen, Carlsbad, CA, USA). cDNA was obtained by reverse transcription with reverse transcription kit (Hitachi, Ltd., Tokyo, Japan). qRT-PCR was employed on the RT-PCR system (StepOneTM, Applied Biosystems, Darmstadt, Germany) with SYBR Premix Ex Taq II (TaKaRa, Dalian, China). U6 and β-actin were taken as internal references, and the relative expressions of circ_0000282, miR-192 and XIAP were calculated by 2^−ΔΔCT^ method. The primer sequences were shown in Table 1.

**Table 1:** Sequences used for qRT-PCR

Name	Primer sequences
circ_0000282	Forward: 5′-CCCTCTGGAATACAACACTGC-3′
	Reverse: 5′-ACCTGTGGGGATGGACATT-3′
miR-192	Forward:5′- GCGGCGGCTGACCTATGAATTG −3’
	Reverse:5′- ATCCAGTGCAGGGTCCGAGG-3′
U6	Forward:5′- TCCGATCGTGAAGCGTTC-3’
	Reverse:5′- GTGCAGGGTCCGAGGT −3’
XIAP	Forward: 5′- GACAGTATGCAAGATGAGTCAAGTCA −3′
	Reverse: 5′-GCAAAGCTTCTCCTCTTGCAG −3′
β-actin	Forward:5′- CACCCCGTGCTGCTGACCGAGGCC-3’
	Reverse:5′-CCACACGGAGTACTTGCGCTCAGG −3’

### Cell counting kit-8 (CCK-8) assay

Transfected cells (1 × 10^3^ / well) were inoculated into 96-well plates with 3 replicated wells in each group. After the cells adhered to the wall, 90 μL of medium and 10 μL of CCK-8 solution (Dojindo Molecular Technologies, Kumamoto, Japan) were added into each well, and a blank control well containing only medium and CCK-8 solution was set up. After incubation for 1 h, the optical density (OD) values of each well were determined with a microplate reader (Bio-Rad Laboratories, Inc., Hercules, CA, USA). With the same method, OD values at 450 nm were detected at 24 h, 48 h, 72 h and 96 h.

### BrdU assay

Cells (1 × 10^5^/mL) were inoculated into a 35 mm diameter culture plate (with a cover slip placed inside), cultured for 1 d, then 1.0 mg/mL of BrdU solution (Applied Biosystems, Foster City, CA, USA) was added, and the cells were incubated at 37 °C for 4 h, and then the culture solution was discarded. The slip was rinsed with PBS for 3 times and the cells were fixed with methanol for 10 min. After the slide was dried, endogenous oxides were inactivated, the unspecific antigens were blocked with 5% rabbit serum. After DNA was denatured, the cells were rinsed with PBS and the primary antibody was added. Moreover, the cells were incubated with secondary antibodies for 1 h. Subsequently, the cells were stained with DAPI staining solution (Beyotime, Shanghai, China). BrdU positive cells in the 3 fields of view were randomly counted under the microscope, and the average value was taken.

### Western blot

RIPA buffer (Beyotime, Shanghai, China) was employed to lyse cells to obtain total protein, and supernatant was collected after centrifugation. The protein concentration was evaluated by BCA protein assay kit (Beyotime, Shanghai, China). After denaturation, the protein samples were separated by 12% sodium dodecyl sulfate polyacrylamide gel electrophoresis and transferred to polyvinylidene fluoride (PVDF) membrane (EMD Millipore Corp, Bedford, MA, USA). 5% defatted milk was used to block the membrane at room temperature for 2 h and then the the membrane was incubated with primary anti-XIAP antibody (1: 1000, ab28151, Abcam, Cambridge, UK), anti-Bax antibody (1:1000, ab32503, Abcam, Cambridge, UK) and anti-Bcl-2 antibody (1, 1000, ab196495, Abcam, Cambridge, UK) at 4 °C overnight. The next day, after the membrane was washed by TBST buffer, the membrane was incubated with HRP labeled secondary antibody (Beyotime, Shanghai, China) for 1 h at room temperature. After the membrane was washed by TBST buffer again, the protein bands were visualized by electrochemical luminescence (ECL) kit (Tanon, Shanghai, China).

### Flow cytometric analysis

Cells were collected and washed with cold phosphate buffered saline (PBS) buffer, and then fixed in 70% ethanol at 4 °C for at 30 min, rinsed twice in cold PBS, then suspended in PBS containing 2 μg/mL RNase (Sigma-Aldrich, Louis, Mo, USA), and then incubated at 37 °C for 30 min, and then stained with propidium iodide staining solution (BD Biosciences, San Jose, CA, USA) and Annexin V-FITC kit (Sigma-Aldrich, Louis, Mo, USA) at room temperature for 30 min. After that, FACSalibur Cytometer (Becton Dickinson, San Jose, CA, USA) was employed for apoptosis analysis.

### Dual luciferase reporter assay

Wild-type (wt) circ_0000282 and mutant (mut) circ_0000282 sequences, and wt and mut sequences from the 3′-untranslated region (3′ - UTR) of XIAP were synthesized and inserted into pGL3-basic reporter vectors (Gene-Crea, Wuhan, China). Then HEK-293 cells (5 × 10^3^) were inoculated into a 24-well plate, and HEK-293 cells were transfected with the reporter vectors, together with miR-192 mimics or miR-NC, respectively. After 48 h of transfection, luciferase activity was measured by dual-luciferase report kit (Promega, Madison, WI, USA). Relative firefly luciferase activity was normalized to the renilla luciferase as internal control.

### RNA immunoprecipitation (RIP) assay

In accordance with manufacturer’s instructions, RIP experiments were carried out using Magna RIP RNA-Binding Protein Immunoprecipitation Kit (Millipore, Billerica, Ma, USA). Ago2 plasmid or vector was transfected into HEK-293 cells. Then, 1 × 10^7^ cells were suspended in 100 μL RIP lysis buffer. Cell lysate (200 μL) was incubated with 5 μg of magnetic beads-coupled anti-Ago2 or anti-IgG antibody (Beyotime, Shanghai, China) at 4 °C and rotated overnight. After incubated with protease K buffer, the immunoprecipitated RNA was extracted using RNasy MineElute Cleanup Kit (Genescript Co, Nanjing, China), and reversely transcribed using Prime-Script RT Master Mix (Genescript Co, Nanjing, China). Ultimately, the abundance of circ_0000282 and miR-192 was detected by qRT-PCR.

### Statistical analysis

All data in this study were expressed by mean ± standard deviation (x ± s) and analyzed statistically by SPSS 17.0 (IBM SPSS, Armonk, NY, USA). The difference between the two groups was analyzed by *t*-test. The data of three or more groups were analyzed by one-way ANOVA. *P <* 0.05 meant that the difference was of statistical significance.

## Results

### The expression of circ_0000282 was up-regulated in human OSA tissues and cell lines

**Fig. 1:**
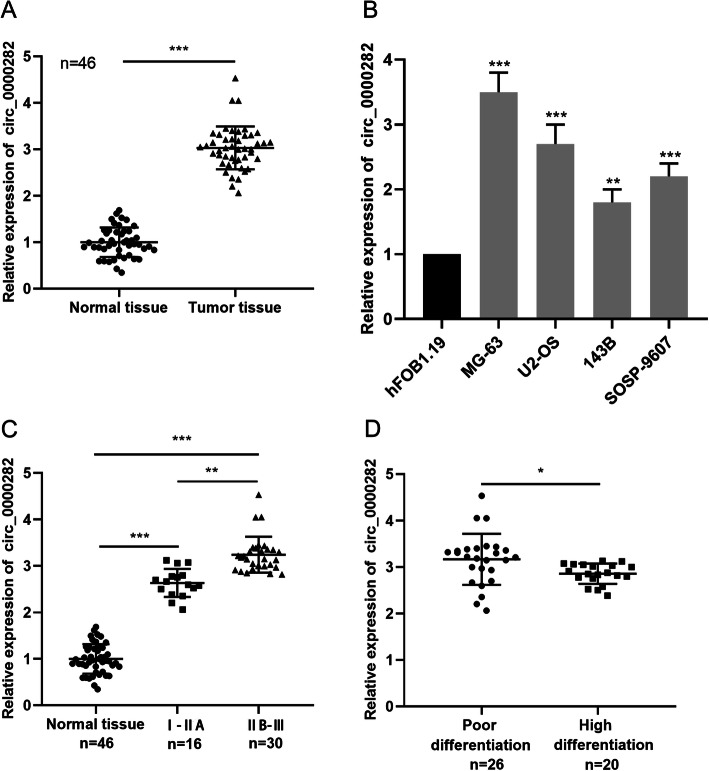
Circ_0000282 was highly expressed in OSA. **a** qRT-PCR was used to detect the expression level of circ_0000282 in human OSA tissues and normal tissues adjacent to cancer. **b** qRT-PCR was used to detect the expression level of circ_0000282 in normal osteoblasts and OSA cell lines. **c**-**d** The correlations between circ_0000282 and OSA clinicopathological features were analyzed. * denotes *P <* 0.05, ** denotes *P <* 0.01, and *** denotes *P <* 0.001

First, the expression of circ_0000282 in cancer tissues and normal tissues of 46 patients with OSA was assessed by qRT-PCR, and it manifested that the expression of circ_0000282 in cancer tissues was remarkably up-regulated in comparison with normal tissues (Fig. 1a). Besides, the expression of circ_0000282 in OSA cell lines (MG-63, U2-OS, 143B and SOSP-9607) was noticeably higher than that of normal osteoblasts cell line (hFOB1.19). (Fig. 1b). Additionally, after analyzing the correlation between the expression of circ_0000282 and clinical pathological features, we demonstrated that the up-regulation of circ_0000282 was markedly associated with higher Enneking stage and the lower degree of tumor differentiation (Fig. 1c-d). In light of these observations, it was concluded that circ_0000282 might function as an oncogene in OSA.

### Circ_0000282 accelerated OSA cell proliferation and suppressed apoptosis

**Fig. 2:**
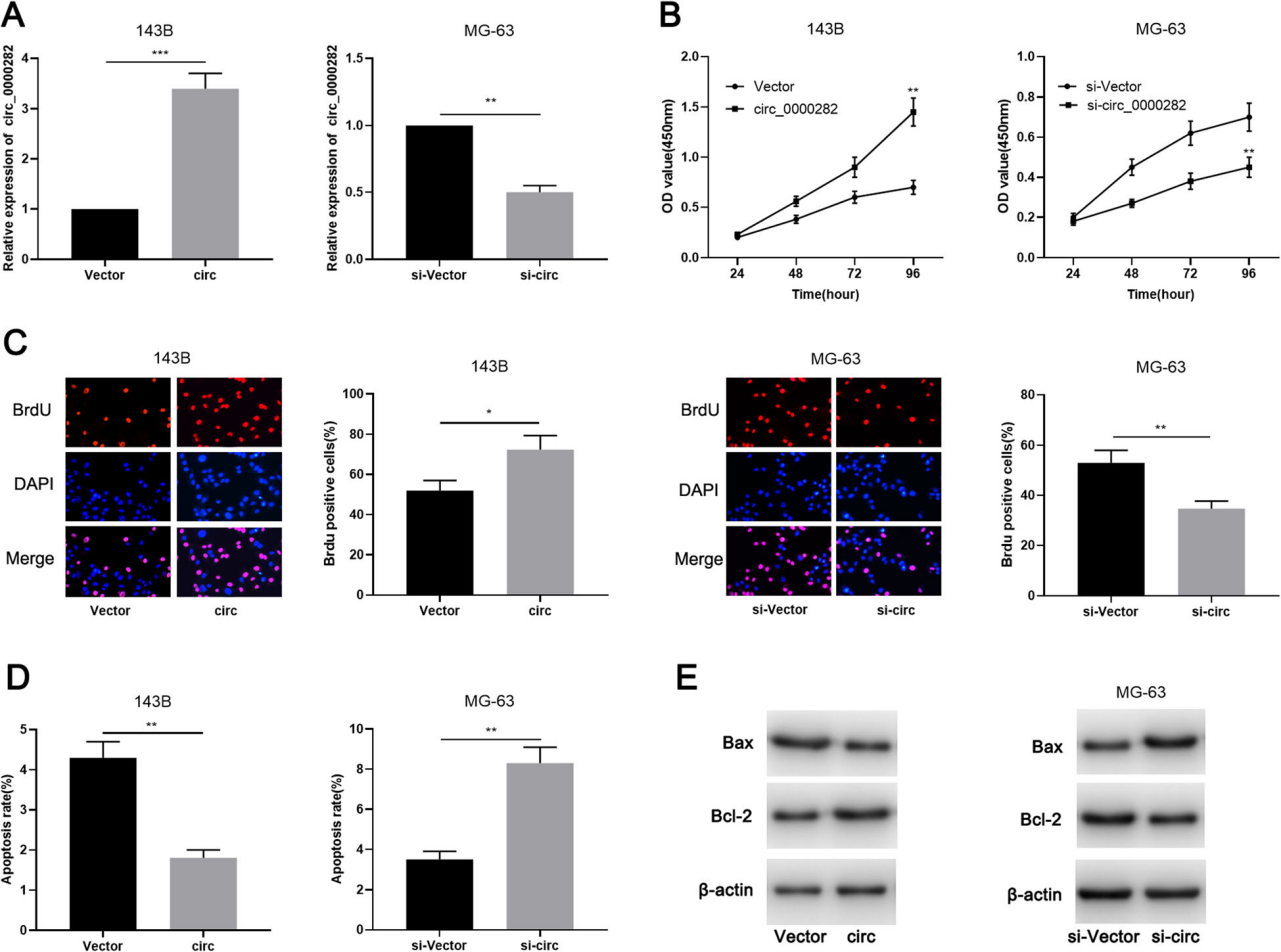
Circ_0000282 promoted OSA cell proliferation and inhibited cell apoptosis. **a** qRT-PCR was used to confirm the successful construction of circ_0000282 overexpression model and circ_0000282 knockdown model. **b-c** The proliferation of OSA cells was detected by CCK-8 experiment and BrdU experiment. **d** Flow cytometry was used to detect the apoptosis of OSA cells. **e** Western blot was used to detect the expressions of XIAP, Bcl-2 and Bax proteins. * denotes *P <* 0.05, ** denotes *P <* 0.01, and *** denotes *P <* 0.001

Considering that the expression of circ_0000282 was lowest in 143B cells, and highest in MG-63 cells among the OSA cell lines, pcDNA3.1-circ_0000282 vector was transfected into 143B cells to establish a circ_0000282 overexpression cell model and circ_0000282 siRNA was transfected into MG-63 cells to establish a circ_0000282 knockdown cell model, respectively. The establishment of the models was then confirmed to be successful by qRT-PCR (Fig. 2a). CCK-8 and BrdU experiments showed that overexpression of circ_0000282 markedly enhanced the proliferation of 143B cells in contrast to control group (Fig. 2b-c) and flow cytometry analysis indicated that overexpression of circ_0000282 remarkably constrained OSA cell apoptosis (Fig. 2d). Western blot experiment showed that overexpression of circ_0000282 significantly increased the expression of Bcl-2 and impeded the expression of Bax (Fig. 2e; Supplementary Fig. 1a). Moreover, knocking down circ_0000282 in MG-63 cells exhibited the opposite effects (Fig. 2b-e; Supplementary Fig. 1a). These findings suggested that circ_0000282 improved OSA cell proliferation and repressed apoptosis.

### Circ_0000282 targetedly regulated miR-192

**Fig. 3:**
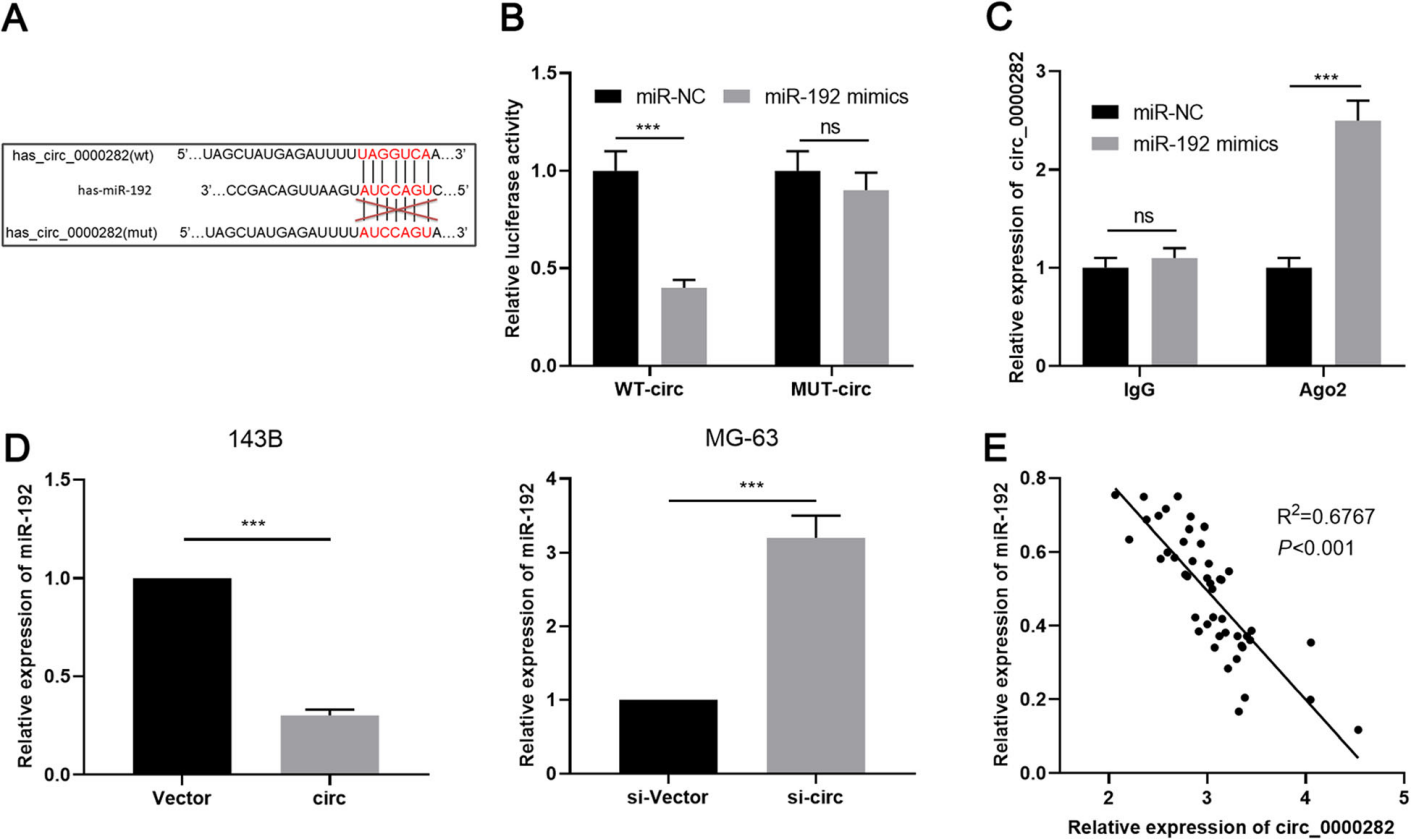
MiR-192 was the target of circ_0000282. **a** Bioinformatics was used to predict the binding site between circ_0000282 and miR-192. **b-c** Dual-luciferase reporter gene assay and RIP experiments proved that circ_0000282 adsorbed miR-192. **d** qRT-PCR was used to detect the effect of circ_0000282 overexpression or knockdown on miR-192 expression. **e** The correlation between circ_0000282 expression and miR-192 expression in OSA samples was analyzed. ns denotes no statistical significance, *** denotes *P* < 0.001

Next, we predicted the downstream target of circ_0000282 through bioinformatics analysis (https://circinteractome.nia.nih.gov/) and noticed a probable binding site existing between cicr_0000282 and miR-192 (Fig. 3a). Dual-luciferase reporter gene assay revealed that miR-192 mimics markedly diminished the luciferase activity of wild type circ_0000282 reporter, yet no remarkable reduction in the luciferase activity of mutant circ_0000282 reporter was observed (Fig. 3b). Consistently, RIP experiments manifested that circ_0000282 and miR-192 were markedly enriched in the immunoprecipitate containing Ago2 (Fig. 3c). Additionally, overexpression of circ_0000282 weaken the expression of miR-192 in 143B cells, while knocking down circ_0000282 promoted the expression of miR-192 in MG-63 cells (Fig. 3d). Additionally, our data displayed that the expression of miR-192 had a negative correlation with the expression of circ_0000282 in OSA tissues (Fig. 3e). All of these data illustrated that circ_0000282 absorbed miR-192 and suppressed miR-192 expression in OSA.

### MiR-192 was lowly expressed in OSA and it targeted XIAP

**Fig. 4:**
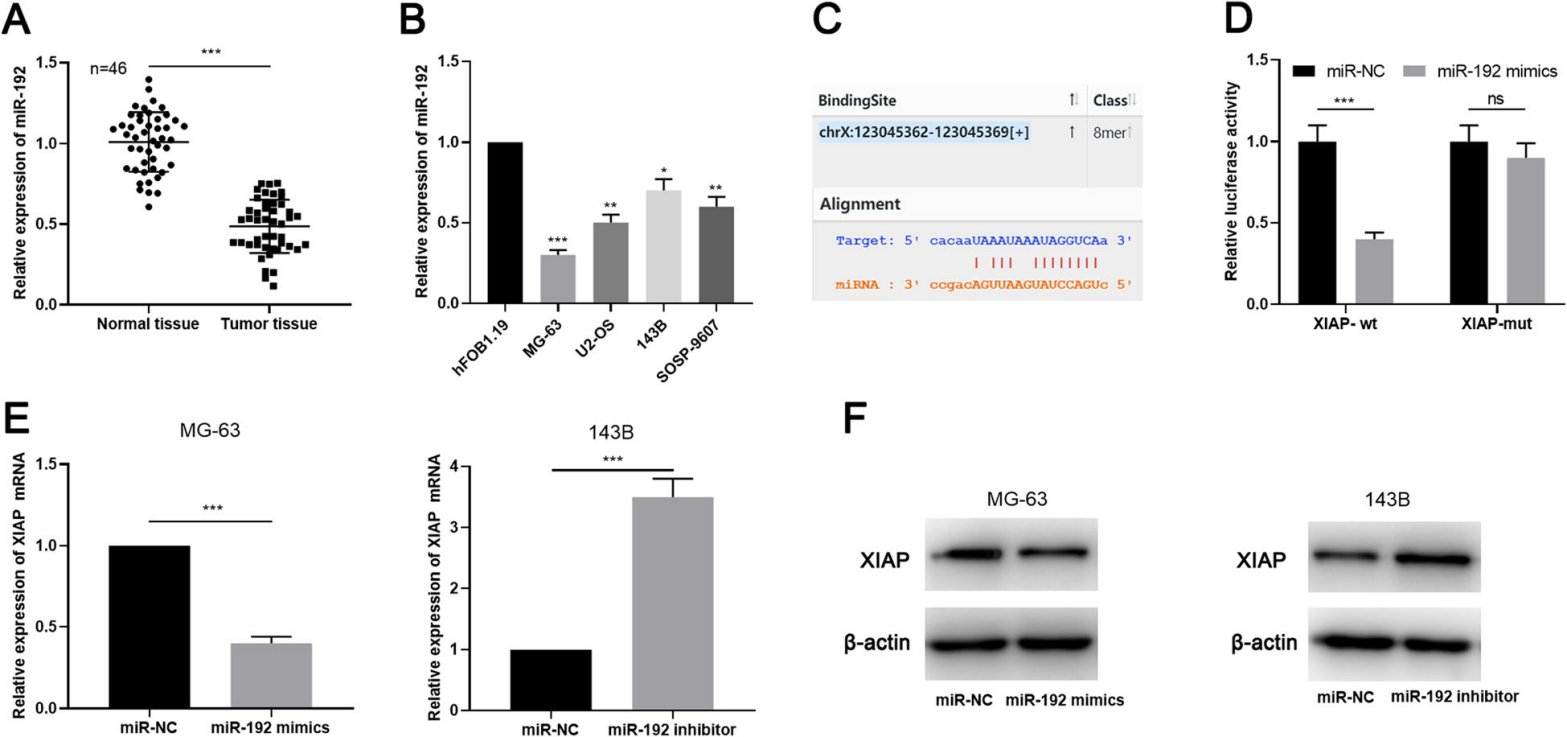
MiR-192 targetedly regulated XIAP. **a**-**b** qRT-PCR was used to detect the expression of miR-192 in OSA tissues and cell lines. **c** Bioinformatics analysis predicted that XIAP was a downstream targets of miR-192. **d** Dual-luciferase reporter gene assay was used to verify the targeting relationship between miR-192 and XIAP. E-F. The expression of XIAP was detected by qRT-PCR and Western blot after miR-192 was selectively regulated in OSA cells. * denotes *P <* 0.05, ** denotes *P <* 0.01, and *** denotes *P <* 0.001

Subsequently, the expression of miR-192 in OSA tissues and cell lines was assessed by qRT-PCR, the findings of which manifested that miR-192 was down-regulated in OSA tissues and cell lines (Fig. 4a-b). Through bioinformatics analysis (http://StarBase.sysu.edu.cn/index.php), a potential binding site between miR-192 and XIAP was noted (Fig. 4c). Dual-luciferase reporter displayed that miR-192 mimics was capable of repressing the luciferase activity of wild type XIAP reporter, but miR-192 mimics didn’t change the luciferase activity of the mutant XIAP reporter (Fig. 4d). Moreover, qRT-PCR and Western blot proved that miR-192 notably suppressed the expressions of XIAP mRNA and protein in OSA cells. Besides, miR-192 inhibitors increased the expression of XIAP mRNA and protein levels (Fig. 4e-f; Supplementary Fig. 1b).

### Circ_0000282 promoted the progression of OSA by regulating miR-192/XIAP axis

**Fig. 5:**
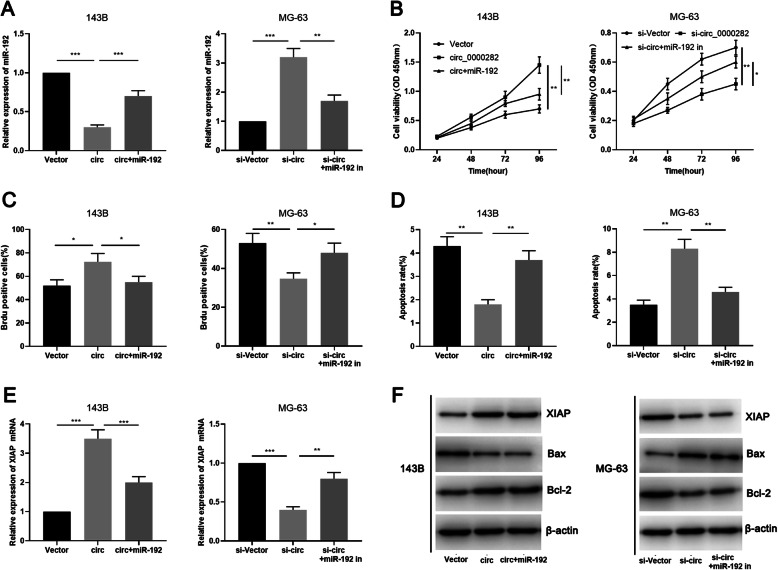
Circ_0000282 promoted progress of OSA by regulating miR-192/XIAP Axis. **a** MiR-192 mimics or miR-192 inhibitors were transfected respectively into OSA cells with circ_0000282 overexpression or knockdown, and the expression level of miR-192 in OSA cells was detected by qRT-PCR. **b-c** The proliferation of OSA cells was detected by CCK-8 experiment and BrdU experiment. **d** Flow cytometry was used to detect the apoptosis of OSA cells. **e** qRT-PCR was used to detect the expression of XIAP mRNA. **f** Western blot was used to detect the expression of XIAP, Bcl-2 and Bax proteins in 143B cells and MG-63 cells. * denotes *P <* 0.05, ** denotes *P <* 0.01, and *** denotes *P <* 0.001

To further validate the regulatory mechanism of circ_0000282/miR-192 axis in OSA, we transfected miR-192 mimics into 143B cells with overexpressed circ_0000282, and transfected miR-192 inhibitors into MG-63 cells with circ_0000282 knockdown (Fig. 5a). Based on this, CCK-8, BrdU, flow cytometry and Western blot were employed to assess OSA cell proliferation, apoptosis and expressions of apoptosis-related proteins Bcl-2, Bax and XIAP. As shown, up-regulation of miR-192 partially reversed the promotion of proliferation of 143B cells due to circ_0000282 overexpression, and partially alleviated the inhibition of apoptosis in 143B cells induced by circ_0000282 (Fig. 5b-d). Moreover, the inhibitory effect on malignant biological behaviors of MG-63 cells caused by circ_0000282 knockdown was partially offset by miR-192 inhibitors (Fig. 5b-d). Additionally, overexpression of circ_0000282 was observed to improve the expression of XIAP and Bcl-2 in 143B cells and repress the expression of Bax; besides, the aforementioned effects were partially weakened after transfection of miR-192 (Fig. 5e-f; Supplementary Fig. 1c). Moreover, transfection of miR-192 inhibitors in MG-63 cells with circ_0000282 knockdown exhibited the reversed effect (Fig. 5e-f; Supplementary Fig. 1c). These results suggested that the regulatory function of circ_0000282 on OSA cells were partly dependent on miR-192/XIAP axis.

## Discussion

Clarifying the mechanism of tumorigenesis and cancer progression can provides useful clues for the diagnosis and treatment of cancer [24–29]. A lot of researches have indicated that circRNAs exhibit a crucial effect on the occurrence and development of human diseases and show potential to be novel markers for detecting diseases, human cancer included [30, 31]. CircRNA can interact with RNA binding proteins (RBPs) to regulate their function to modulate cancer progression [32–35]. In addition, many circRNA have been proved to be involved in post-transcriptional regulation by acting as miRNA’s “sponge”, thus reducing the ability of miRNA to target mRNA [17–19, 35, 36]. Recent studies have proved that hundreds of circRNAs are dysregulated in various types of cancers [37]. A previous study reports 8 up-regulated and 102 down-regulated circRNAs in OSA through bioinformatics analysis [8]. It is reported that circ_0081001 is remarkably up-regulated in OSA cell line, tissue and serum, and its high expression has relation with short overall survival time of the patients [38]. The low expression of circHIPK3 is associated with unfavorable prognosis of OSA patients, and circHIPK3 overexpression remarkably represses OSA cell proliferation, migration and invasion in vitro [39]. These findings indicate that circRNAs play a crucial role in promoting or inhibiting OSA pathogenesis. In this study, we reported for the first time that circ_0000282 was up-regulated in OSA tissues and cell lines, and its high expression level was markedly related to the increase of Enneking stage and low differentiation degree of tumor. Functional experiments manifested that circ_0000282 regulated the proliferation and apoptosis of OSA cells. These data suggested that circ_0000282 played a role in promoting OSA progression.

As a member of miRNA family, miR-192 acts as oncomiR or tumor suppressor in different tumors. For instance, miR-192 is down-regulated in non-small cell lung cancer and it represses the proliferation and epithelial-mesenchymal transition of cancer cells [40]. Likewise, miR-192 represses the bone metastasis of lung cancer by targeting TRIM44 [41]. Conversely, miR-192 expression is up-regulated in prostate cancer and it expedites cancer cell proliferation [42]. miR-192 enhances epithelial-mesenchymal transition of gastric cancer by inhibiting SMG-1 and inactivating Wnt signaling pathway [43]. Previous studies have reported that miR-192 is down-regulated in OSA tissues and cell lines, and it suppresses OSA cell growth, invasion and accelerating cancer cell apoptosis [44]. In the present study, miR-192 was demonstrated to show low expression in OSA tissues and cells, supporting that miR-192 served as a tumor suppressor gene in OSA, which is consistent with the previous study. CircRNA can function as ceRNA to regulate miRNA expression and cancer progression. For example, circ_001350 facilitates proliferation and metastasis of glioma cells and suppresses apoptosis through sponging miR-1236 [45]. In the present work, through bioinformatics analysis, a binding site was noticed to exist between circ_0000282 and miR-192. Dual luciferase reporter gene assay and RIP experiment proved that circ_0000282 was capable of adsorbing miR-192. Additionally, knocking down circ_0000282 increased the expression of miR-192 in OSA cells. Furthermore, miR-192 mimics reversed the promotion of overexpressed circ_0000282 on the malignant biological behaviors of OSA cells. Collectively, these data suggested that the overexpression of circ_0000282 in OSA contributed to miR-192 dysregulation, and miR-192 was a downstream effector of circ_0000282.

Among members of IAP family, XIAP possesses the strongest anti-apoptotic activity [46]. XIAP contains baculovirus IAP repeat (BIR) domains and RING zinc finger motif. BIR domains is able to suppress the functions of caspase-3 and caspase-7, two members of the caspase family of cell-death proteases, while RING domain is able to repress the functions of caspase-9 [46–48]. XIAP protein affects the ratio of Bcl-2/Bax and Bcl-xl/Bax, which play decisive roles in cell death [49]. Another significant impact of XIAP on cancer biology is to activate NF-κB signal pathway. For example, XIAP induces transcription of Beclin1 by activating NFκB signaling pathway to regulate autophagy [50]. XIAP exerts an oncogenic role in multiple cancers. For example, XIAP has high expression in bladder cancer tissues and it accelerates disease progression [51]. XIAP also represses apoptosis of prostate cancer cells and glioblastoma cells [52, 53]. In this study, we predicted and verified that XIAP was a downstream target of miR-192 in OSA. miR-192 was proved to suppress the expression of XIAP mRNA and protein levels, and circ_0000105 was capable of reducing the expression of miR-192 and indirectly up-regulating the expression of XIAP. These findings demonstrated that the circ_0000282/ miR-192/XIAP axis was involved in regulating OSA progression.

## Conclusions

Exploring the molecular mechanisms of cancer progression can provide new insights for cancer treatment [54, 55]. Our study demonstrates that circ_0000282 is up-regulated in OSA tissues and cell lines, and its high expression is probably associated with unfavorable prognosis of OSA patients. In the view of mechanism, we confirm that circ_0000282 promotes OSA cell proliferation and suppresses apoptosis through miR-192/ XIAP axis. This novel ceRNA network offers potential diagnostic biomarkers and therapy targets for OSA.

## Supplementary information

**Additional file 1: Supplementary Figure 1.** Densitometric analysis of western blots.

## Data Availability

The data used to support the findings of this study are available from the corresponding author upon request.

## Abbreviations

CircRNAs: Circular RNAs
OSA: Osteosarcoma
qRT-PCR: Quantitative real-time polymerase chain reaction
XIAP: X-linked inhibitor of apoptosis protein
Bcl-2: B-cell lymphoma-2
CCK-8: Cell Counting Kit-8
Bax: Bcl-2 associated X protein
BrdU: Bromodeoxyuridine
RIP: RNA immunoprecipitation
CeRNA: Competitive endogenous RNA
miRNA: Micro RNA
IAP: Inhibitor of apoptosis
DMEM: Dulbecco Modified Eagle Medium
FBS: Fetal bovine serum
SiRNAs: Small interference RNAs
PVDF: Polyvinylidene fluoride
ECL: Eelectrochemical luminescence
PBS: Phosphate buffered saline
BIR: Baculovirus IAP repeat
