# Supplementary figures and images for: Circular RNA hsa_circ_0000282 contributes to osteosarcoma cell proliferation by regulating miR-192/XIAP axis
Source: BMC Cancer. 2020 Oct 23;20:1026. doi: 10.1186/s12885-020-07515-8 (PMC7583201; doi:10.1186/s12885-020-07515-8)

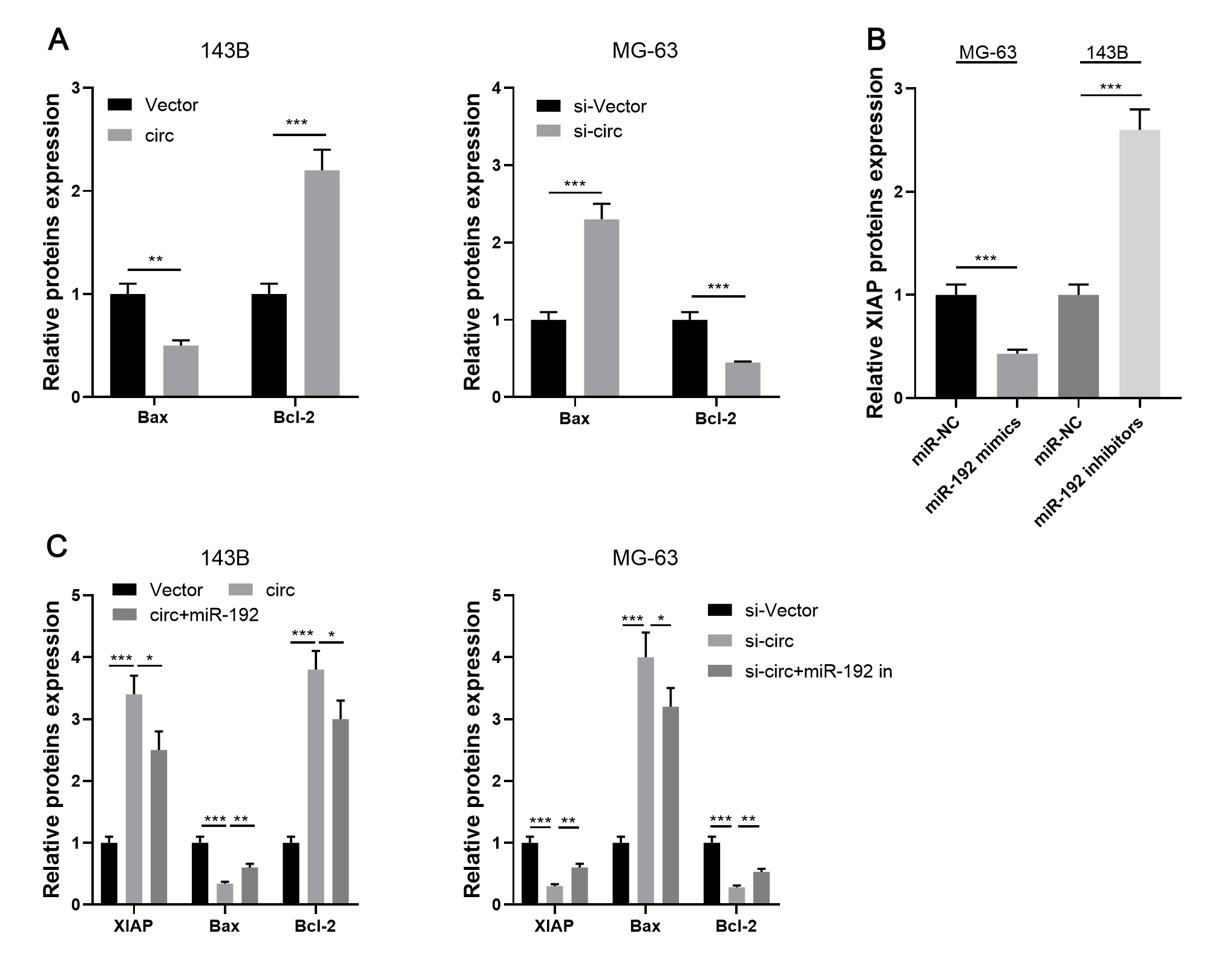

Supplement: Supplementary file 1 — Additional file 1: Supplementary Figure 1. Densitometric analysis of western blots. [file 12885_2020_7515_MOESM1_ESM.tif]
